# Rapid and precise detection of cancers via label-free SERS and deep learning

**DOI:** 10.1007/s00216-023-04730-7

**Published:** 2023-05-17

**Authors:** Chang-Chun Xiong, Shan-Shan Zhu, Deng-Hui Yan, Yu-Dong Yao, Zhe Zhang, Guo-Jun Zhang, Shuo Chen

**Affiliations:** 1 Research Institute of Medical and Biological Engineering, Ningbo University, Ningbo, 315211 China; 2 Faculty of Electrical Engineering and Computer Science, Ningbo University, Ningbo, 315211 China; 3 Health Science Center, Ningbo University, Ningbo, 315211 China; 4 Department of Urology, First Affiliated Hospital, China Medical University, Shenyang, 110001 China; 5 Department of Hematology, Shengjing Hospital, China Medical University, Shenyang, 110022 China; 6 College of Medicine and Biological Information Engineering, Northeastern University, Shenyang, 110169 China

**Keywords:** Surface-enhanced Raman scattering (SERS), Deep learning, Cancer detection, Gradient-weighted class activation mapping (Grad-CAM)

## Abstract

Early, express, and reliable detection of cancer can provide a favorable prognosis and decrease mortality. Tumor biomarkers have been proven to be closely related to tumor occurrence and development. Conventional tumor biomarker detection based on genomic, proteomic, and metabolomic methods is time and equipment-consuming and always needs a specific target marker. Surface-enhanced Raman scattering (SERS), as a non-invasive ultrasensitive and label-free vibrational spectroscopy technique, can detect cancer-related biomedical changes in biofluids. In this paper, 110 serum samples were collected from 30 healthy controls and 80 cancer patients (including 30 bladder cancer (BC), 30 adrenal cancer (AC), and 20 acute myeloid leukemia (AML)). One microliter of blood serum was mixed with 1 μl silver colloid and then was air-dried for SERS measurements. After spectral data augmentation, one-dimensional convolutional neural network (1D-CNN) was proposed for precise and rapid identification of healthy and three different cancers with high accuracy of 98.27%. After gradient-weighted class activation mapping (Grad-CAM) based spectral interpretation, the contributions of SERS peaks corresponding to biochemical substances indicated the most potential biomarkers, i.e., L-tyrosine in bladder cancer; acetoacetate and riboflavin in adrenal cancer and phospholipids, amide-I, and α-Helix in acute myeloid leukemia, which might provide an insight into the mechanism of intelligent diagnosis of different cancers based on label-free serum SERS. The integration of label-free SERS and deep learning has great potential for the rapid, reliable, and non-invasive detection of cancers, which may significantly improve the precise diagnosis in clinical practice.

**Graphical abstract:**

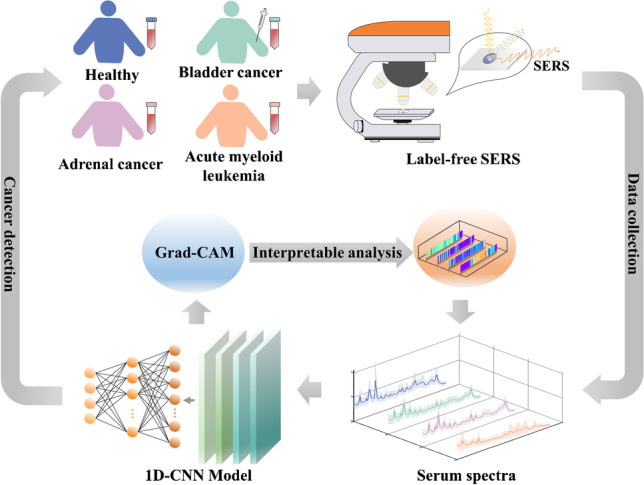

## Introduction

Currently, cancer is a serious threat to people worldwide accompanied by its increasing incidence and mortality rates. In China, an estimated 4.5 million new cases and 3.0 million deaths due to cancer occurred in 2020, and cancer has become the major cause of death among Chinese residents [1]. Early, express, and reliable detection of cancer is closely associated with a favorable prognosis and decreasing mortality.

The conventional methods of cancer detection mainly include imaging examination [2], histopathological analysis [3], and tumor biomarker detection [4]. Although imaging examination is convenient and non-invasive, it heavily relies on the radiologists’ experience and lacks sensitivity to early cancer. In clinical, the golden standard for cancer diagnosis is histopathology, which has advantages of high sensitivity and accuracy, while limits of time-consuming, highly invasive, and intensive sample preparation restrict its wide applications for cancer screening and early diagnosis. In recent years, plenty of biomedical components derived from tumors (i.e., tumor biomarkers) in tissue and biofluids have been proven to be closely related to tumor occurrence and development [5]. Thus, tumor biomarker detection has become a competitive way for early cancer screening and diagnosis. However, traditional tumor biomarker detection performed on a tiny portion of cancer tissue results in an inadequate recognition and trace of one patient’s tumor [6]. Recently, the detection of tumor-derived biomedical substances (such as DNA, RNA, and cancer-related proteins) in biofluids [7] has attracted remarkable attention for its role in early cancer detection with the advantages of non-invasive, reproducible, dynamic monitoring, and overcoming tumor molecular heterogeneity. Now, tumor biomarkers detection is traditionally carried out by genomic [8], proteomic [9], and metabolomics [10] methods. Although the above-mentioned techniques have been well elaborated and can achieve high sensitivity and specificity results, their utilization is time- and equipment-consuming and always need a specific target marker related to the tumor-derived biomedical components. Therefore, establishing a convenient, rapid, sensitive, and label-free method for tumor biomarker detection is still challenging and greatly needed.

Raman spectroscopy is a typical vibrational spectroscopy technique that can provide fingerprint information about the molecular composition and structure of samples through an inelastic scattering phenomenon between vibrating molecules and monochromatic photons [11]. In recent years, Raman spectroscopy has become a powerful tool for the detection of cancers based on different biological samples [12, 13]. However, inherent weak Raman signal hindered its biomedical applications, especially in biofluid detection [14, 15]. Fortunately, when molecules adsorb on metals with rough surfaces, the molecular Raman signal can be significantly amplified, i.e., surface-enhanced Raman scattering (SERS) [16], in which the SERS enhancement effect mainly includes both electromagnetic enhancement effect [17] and chemical enhancement effect [18]. Thus, the SERS technique has recently established itself as a powerful strategy for the detection of cancer-derived changes in biofluids with fast, non-invasive, and label-free characteristics [19–22], which may provide more comprehensive information on various potential tumor biomarkers without any specific target marker [23].

However, spectral analysis of biofluids is often a prerequisite to interpreting the potential information of the spectra. Traditional spectral analysis is usually performed by tedious preprocessing and multivariate statistical methods (such as Savitzky-Golay (SG), genetic algorithm (GA), linear discriminant analysis based on principal component analysis (PCA-LDA), support vector machine based on principal component analysis (PCA-SVM)) [24]; a complicated process is challenging for extracting useful information from the high dimensions of spectra and remains a major bottleneck to achieving high accuracy. As an end-to-end method, deep learning contains automatic feature extraction and classification module, providing the possibility to overcome the above problems. Although typical deep learning approaches (i.e., convolutional neural networks (CNN), recurrent neural networks (RNN), generative adversarial networks (GAN)) have shown superior performance in analyzing spectral signals [25], including SERS in biofluid samples of different cancers [26–30], the diagnosis mechanism of deep learning for spectral analysis based on label-free SERS is still unclear. In clinical practice, it is significant to explore the potential diagnosis mechanism of different cancers in biofluids for precise treatment.

In this paper, 110 serum samples were collected from 30 healthy controls and 80 cancer patients (including 30 bladder cancer (BC), 30 adrenal cancer (AC), and 20 acute myeloid leukemia (AML)). One microliter of blood serum was mixed with 1 μl silver colloid and then was air-dried for SERS measurements. After spectral data augmentation, a one-dimensional convolutional neural network (1D-CNN) was proposed to precisely and rapidly identify healthy and three different cancers. Then, an intelligent and quantitative spectral interpretation using gradient-weighted class activation mapping (Grad-CAM) [31] was performed to objectively localize the spectral regions of interest (ROI), i.e., specific Raman peaks corresponding to biomolecules responsible for the final identification.

## Materials and methods

### Serum preparation

This study included 110 blood serum samples taken from 30 healthy controls and 80 cancer patients (including 30 bladder cancer (BC), 30 adrenal cancer (AC), and 20 acute myeloid leukemia (AML)) from the First Affiliated Hospital and Shengjing Hospital of China Medical University. Cancer patients were diagnosed by histopathology examinations and patients with other systemic physical diseases except for bladder cancer, adrenal cancer, and acute myeloid leukemia were excluded. The human blood study was supported and approved by the medical ethics committee of the First Affiliated Hospital and Shengjing Hospital of China Medical University, and all participants have signed the relevant informed consent forms. To ensure the serum samples accurately reflect the physiological conditions of the participants, each participant was asked to fast overnight and then 3 ml peripheral blood was collected from them between 7:00 to 8:00 in the morning. To separate the serum from the blood, each blood sample was centrifuged at 3000 rpm for 10 min. The obtained serum samples were then immediately stored in a freezer at  − 80 ℃ until SERS collection was performed.

### SERS measurements

Before SERS measurements, 1 μl each of thawed serum sample and 1 μl silver colloid were mixed homogenously and incubated for 2 h at room temperature. In particular, the silver colloid was prepared by reducing silver nitrate with trisodium citrate according to the Lee-Meisel method in our previous studies [20].

For SERS measurements, a 1 μl mixture of serum and silver colloid was dropped on the aluminum substrate, and air-dried mixture sample was finally observed by a confocal Raman microscope (HR Evolution, Horiba JY, France). In detail, a 785 nm diode laser was used as the excitation source and the laser delivered a power of 4.3 mW to the sample through a 20 × objective lens (numerical aperture = 0.4, diameter of laser spot = 2.4 μm). The spectra were recorded in the 400–1800 cm^−1^ Raman shift range with a 2 cm^−1^ spectral resolution. Each spectrum was cumulatively acquired once with an integration time of 10 s. Five measurements were obtained from five randomly selected locations including the central and edge region of each serum sample, and the average of these five measurements was used as the final SERS spectrum for further processing and analysis.

### Data preprocessing

In this study, a total of 110 serum SERS spectra were obtained from different groups (i.e., 30 healthy controls, 30 bladder cancer, 30 adrenal cancer, and 20 acute myeloid leukemia).

For deep learning analysis, data preprocessing was performed by the following steps. Firstly, 110 SERS spectra were randomly divided into 5 sets, with 22 spectra in each set. One set served as a test dataset, one set served as a validation dataset, and the remaining three sets served as a training dataset. In order to avoid the overfitting problem resulting from a relatively small number of samples, a data augmentation method of random linear combination [32] of three spectra in the same group was employed to generate new SERS spectra, in which the diversity of spectra increased, while the original distribution of each spectrum can be maintained. Thus, 660, 220, and 220 SERS spectra were used as training, validation, and test dataset, respectively. Then, maximum-minimum normalization was performed on all SERS spectra to eliminate the effect caused by the difference in SERS spectral intensity of different serum samples. These preprocessed spectra served as the input of the 1D-CNN model.

For the average spectra comparison and traditional machine learning analysis (i.e., PCA-LDA and PCA-SVM), data preprocessing was performed by the following steps. Firstly, the noise was reduced by the Savitzky-Golay algorithm [33]. Then, a fifth-order polynomial fitting method [34] was used to remove the background interference. Finally, maximum-minimum normalization was performed on each SERS spectrum to reduce the influence of spectral intensity variability for making a better comparison of the spectral characteristics among different groups for multivariate data analysis.

For both deep learning and traditional machine learning methods, five fold cross-validation [35] was taken to objectively evaluate the models.

### 1D-CNN model

**Fig. 1:**
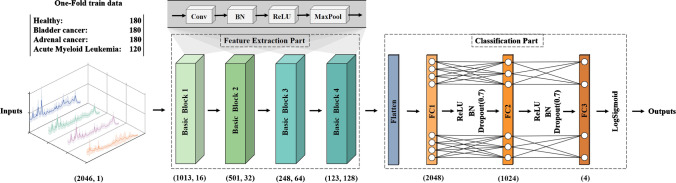
Architecture of the proposed 1D-CNN model

One 1D-CNN model was proposed in this study as shown in Fig. 1, which consisted of two major parts: feature extraction and classification. The feature extraction part included four basic blocks (i.e., basic block1, basic block2, basic block3, and basic block4), and each of them contained a convolutional layer, a normalization layer, an activation layer, and a pooling layer. Specifically, the number and size of convolutional kernels in the four convolutional layers were 16, 32, 64, and 128 and 1 × 21, 1 × 11, 1 × 5, and 1 × 3, respectively. Large convolutional kernels in the shallow layers of the model allowed for a larger perceptual field and a more comprehensive range of global features can be extracted. Small convolutional kernels with smaller perceptual fields in the deep layers enabled much richer characteristic peak information which can be extracted [36]. Batch normalization (BN) was used in the normalization layer after each convolutional layer to avoid the overfitting and vanishing gradient problem [37]. Rectified linear unit (ReLU) [38] activation function was used in the activation layer and max-pooling [39] with a window size of 1 × 2 and stride of 2 in the pooling layer aimed to decline the number of model parameters. The classification part consisted of one flatten layer and three fully connected layers (i.e., FC1, FC2, and FC3). The FC1 and FC2 layers were followed by the ReLU activation layer, BN, and dropout [40] (dropout rate *p* = 0.7) to reduce the occurrence of overfitting and enhanced the generalization ability of the model. Log-Sigmoid function [41] after the FC3 layer was used to achieve the final classification results.

In this model, the one-dimensional spectra with a length of 2046 served as inputs, and the length and dimension were adjusted by the feature extraction part, i.e., basic block1 to basic block4. The numbers below the basic blocks indicated the length and dimension of the output, respectively. After feature extraction, the features were flattened and used as input for the FC1 layer. The number below the FC layers indicated the number of neurons. Finally, 4 class probabilities were outputted by the Log-Sigmoid function.

During the training procedure, Radam [42] was used as the optimizer function to train the model. The learning rate of Radam was set at 1 × 10^−4^, and the batch size was set at 128. Due to the imbalance of data size of each class in the dataset, a weighted cross-entropy loss [43] was adopted as the loss function. Specifically, the class weights were firstly calculated by dividing the number of spectra in each class by the number of total spectra in all classes; the weighted loss was then obtained by multiplying the loss by the corresponding class weight.

### Comparison methods

In order to assess the ability of our model to discriminate the serum SERS spectra in different groups, traditional machine learning methods (i.e., PCA-LDA, PCA-SVM) and classical deep learning models (i.e., CNN including AlexNet, GoogLeNet, VGGNet, ResNet, and DenseNet; RNN; GAN) were performed for a comparative study.

### Model evaluation

In this study, accuracy, precision, recall, and F1-score were selected as important metrics for evaluating the classification ability of models. The equations were as below:

1 $${Accuracy}_{\left(C\right)}=\frac{\left(TP+TN\right)}{(TP+TN+FP+FN)}$$

2 $${Precision}_{\left(C\right)}=\frac{TP}{(TP+FP)}$$

3 $${Recall}_{\left(C\right)}=\frac{TP}{(TP+FN)}$$

4 $${F1-score}_{\left(C\right)}=\frac{{2\times Precision}_{\left(C\right)}\times {Recall}_{\left(C\right)}}{{Precision}_{\left(C\right)}+{Recall}_{\left(C\right)}}$$

where $TP$, $FP$, $TN$, and $FN$ represented true positive, false positive, true negative, and false negative for class $C$ samples, respectively, in which $C$ = {healthy controls, bladder cancer, adrenal cancer, acute myeloid leukemia}.

In addition, the receiver operating characteristic (ROC) curve was further plotted for the corresponding test results, and the area under the ROC curve (AUC) was calculated for each of the four groups below:

1. Healthy controls versus cancer;
2. Bladder cancer versus non-bladder cancer;
3. Adrenal cancer versus non-adrenal cancer;
4. Acute myeloid leukemia versus non-acute myeloid leukemia.

### Spectral interpreting analysis method

**Fig. 2:**
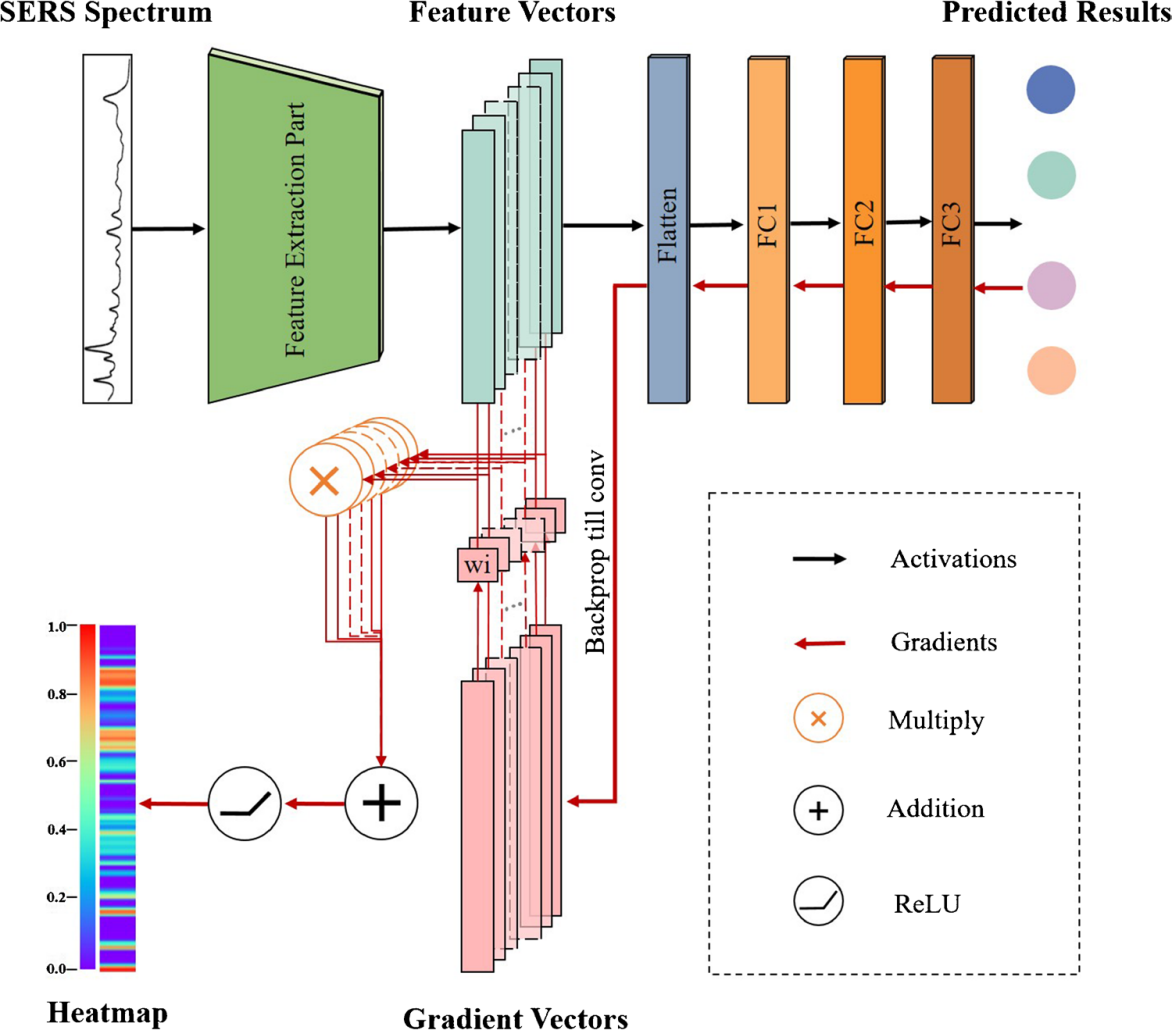
Spectral interpretable analysis based on Grad-CAM method

After the classification of serum SERS spectra from four different groups (i.e., healthy controls, bladder cancer, adrenal cancer, and acute myeloid leukemia), an intelligent and quantitative spectral interpretation method based on Grad-CAM method [31] was performed to objectively localize the spectral ROI, i.e., specific Raman peaks corresponding to biomolecules responsible for the final identification making. As shown in Fig. 2, the gradients corresponding to a predicted class were back-propagated until the last convolutional layer of the model and then global-averaged in each channel to obtain the weight ${w}_{\mathrm{i}}$ of the feature vector in this channel. Then, the feature vectors were weighted summation and activated by the ReLU function to achieve a coarse heatmap along the wavenumber dimension. The heatmap indicated the contribution degree of each wavelength of the input SERS spectrum, i.e., specific Raman peaks corresponding to biomolecules responsible for the final classification making, in which the red color represented a high contribution while the purple color represented a low contribution to the final classification result. And then, the contribution degree ${C}_{i}$ of the prominent Raman peaks for the final classification making was quantified by Eq. 5.

5 $${C}_{i}=\frac{{S}_{i}}{\sum_{i=1}^{n}{S}_{i}}$$

where $n$ is the total number of all prominent Raman peaks and ${S}_{i}$ is the area under full width at half maximum intensity of the *i*th prominent Raman peak.

## Results and discussions

### Prominent SERS peaks

**Fig. 3:**
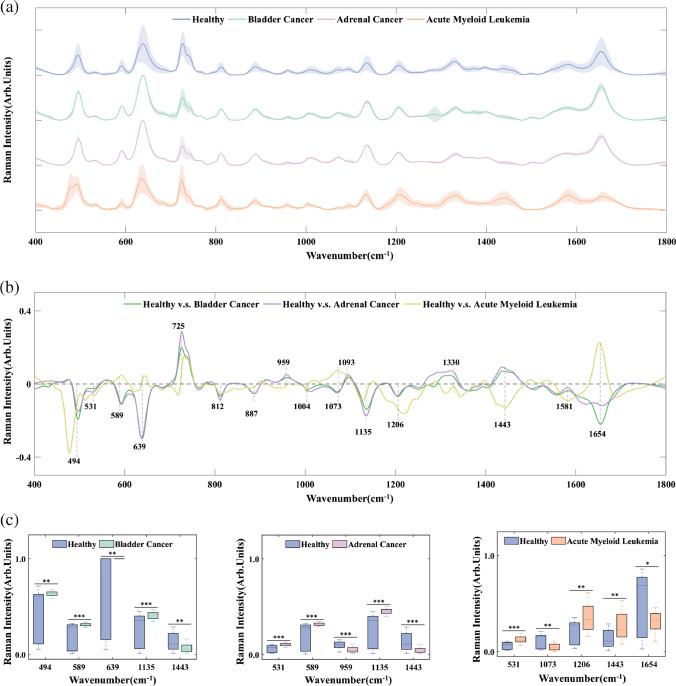
**a** The average SERS spectra after preprocessing from healthy controls, bladder cancer, adrenal cancer, and acute myeloid leukemia groups, in which the shaded areas represent the standard deviations among each group; **b** the subtracted spectra between healthy controls and different cancer groups; **c** the boxplots of Raman intensities at five peaks with most statistically significant differences between healthy controls and bladder cancer; healthy controls and adrenal cancer; healthy controls and acute myeloid leukemia, respectively (notes: ***p < 0.0001; **p < 0.001; *p < 0.01)

Figure 3a shows the average serum SERS spectra after preprocessing from healthy controls, bladder cancer, adrenal cancer, and acute myeloid leukemia groups. It can be seen that the prominent SERS peaks of healthy control and cancer groups are observed at 494, 531, 589, 639, 725, 812, 887, 959, 1004, 1073, 1093, 1135, 1206, 1330, 1443, 1581, and 1654 cm^−1^. These prominent SERS peaks can be assigned to corresponding vibrational modes and biochemical substances based on previously published studies [44–49] (as listed in Table 1). Figure 3b shows the subtracted spectra between healthy control and different cancer groups. In addition, the boxplots of five SERS peaks with the most statistically significant differences are selected by a two-sample *t*-test and plotted in Fig. 3c. The bladder cancer group shows a significant increase in SERS intensity of L-arginine (494 cm^−1^), amide-VI (589 cm^−1^), L-tyrosine and lactose (639 cm^−1^), and D-mannos (1135 cm^−1^) compared to the healthy group. However, the SERS intensity of glycine and L-proline (1443 cm^−1^) decreases in the bladder cancer group compared to the healthy group. The adrenal cancer group exhibits more intense Raman signals of riboflavin (531 cm^−1^), amide-VI (589 cm^−1^), and D-mannos (1135 cm^−1^) compared with those in the healthy group. In contrast, less L-valine (959 cm^−1^) and glycine and L-proline (1443 cm^−1^) are observed in the adrenal cancer group. In the acute myeloid leukemia group, the SERS bands of riboflavin (531 cm^−1^), L-tryptophan and phenylalanine (1206 cm^−1^), and glycine and L-proline (1443 cm^−1^) show higher signals than those of healthy group. While lower SERS signals of collagen (1073 cm^−1^), phospholipids, amide-I, and α-Helix (1654 cm^−1^) are found in acute myeloid leukemia groups.

**Table 1:** Assignments of prominent blood serum SERS peaks

Peak position (cm^−1^)	Assignments
494	Ring vibration, cellulose, guanine, L-arginine
531	Cholesterol ester
589	Ascorbic acid, amide-VI
639	C-S stretching vibration, L-tyrosine, lactose
725	C-H bending vibration, adenine, coenzyme A
812	C-C-O stretching vibration, L-serine, glutathione
887	C-O-H bending vibration, glutathione, D-( +)-galactosamine
959	α-Helix, L-proline, L-valine
1004	C-C symmetric stretch, phenylalanine
1073	C-N stretching vibration, collagen
1093	C-N stretching vibration, D-mannos
1135	C-N stretching vibration, D-mannos
1206	Ring vibration, L-tryptophan, phenylalanine
1330	C-H stretching vibration, nucleic acid bases
1443	CH2 bending vibration, glycine, L-proline, stearic acid
1581	C = C bending vibration, phenylalanine, acetoacetate, riboflavin
1654	C = O stretching vibration, phospholipids, amide-I, α-Helix

### Spectral classification

**Fig. 4:**
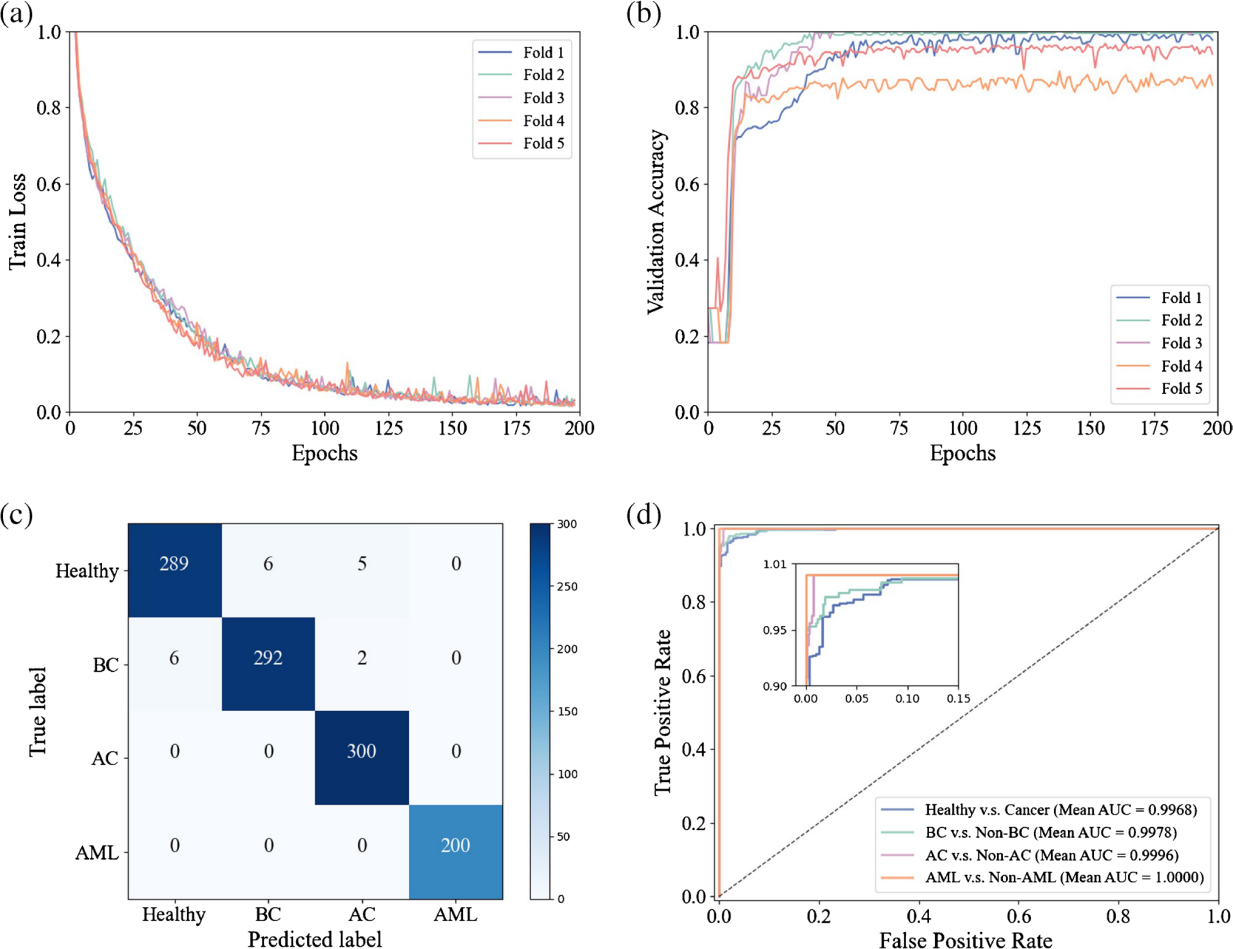
**a** The training loss curves versus different epochs; **b** the accuracy curves on the validation dataset versus different epochs; **c** the confusion matrix on the test dataset after five fold cross-validation; **d** the ROC curves and the AUCs (BC, bladder cancer; AC, adrenal cancer; AML, acute myeloid leukemia)

Figure 4a and b show the training loss and validation accuracy curves versus epochs after the five fold cross-validation. It can be found that the loss curve converges at 200 epochs; in the meantime, the accuracy of the validation dataset is almost stable. In addition, the confusion matrix on the test dataset after the fivefold cross-validation is shown in Fig. 4c. The final accuracy, precision, recall, and F1-score on the test dataset are 98.27%, 98.34%, 98.27%, and 98.27%, respectively. Specifically, 99% of cancer patients are correctly identified, in which 97.33% of bladder cancer patients are correctly identified and 100% of adrenal cancer patients and acute myeloid leukemia patients are correctly identified; these results are consistent with the ROC curves and AUCs plotted in Fig. 4d. The above results demonstrate the excellent performance of our model for identifying serum SERS spectra among various cancers.

**Fig. 5:**
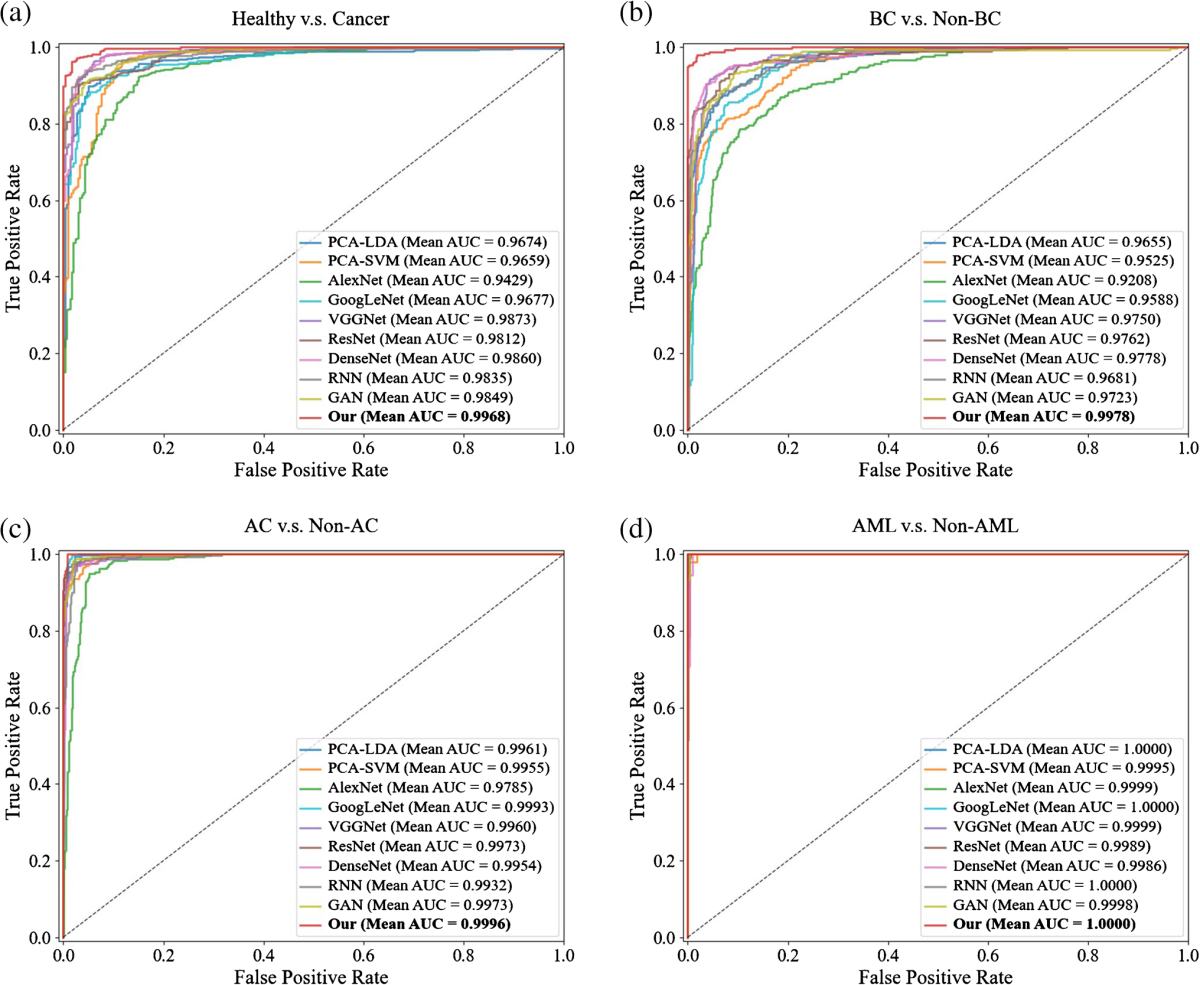
ROC curves and AUCs of different models in each of two groups: **a** healthy versus cancer; **b** BC versus non-BC; **c** AC versus non-AC; **d** AML versus non-AML (BC, bladder cancer; AC, adrenal cancer; AML, acute myeloid leukemia)

For comparison of the classification performance, traditional machine learning methods and other classical deep learning models are adopted to recognize serum SERS spectra among healthy controls, bladder cancer, adrenal cancer, and acute myeloid leukemia groups. The classification results are shown in Table 2, in which the best performance with accuracy is 98.27% obtained by our model. Furthermore, Fig. 5 shows the ROC curves and AUCs of different models on the test dataset in each of the two groups, i.e., healthy controls versus cancer, bladder cancer versus non-bladder cancer, adrenal cancer versus non-adrenal cancer, and acute myeloid leukemia versus non- acute myeloid leukemia. The highest AUCs are found in our model in all these four groups.

**Table 2:** Classification results of serum SERS spectra based on different methods, in which the best result for each indicator among different methods are shown in bold

Method	Accuracy	Precision	Recall	F1-score
PCA-LDA	90.91%	90.43%	90.38%	90.23%
PCA-SVM	90.45%	92.08%	90.45%	90.51%
AlexNet	85.36%	86.33%	85.36%	85.24%
GoogLeNet	90.27%	90.70%	90.27%	90.33%
VGGNet	93.36%	94.05%	93.64%	93.67%
ResNet	93.55%	94.14%	93.45%	93.30%
DenseNet	93.36%	93.91%	93.36%	93.29%
RNN	91.91%	92.48%	91.91%	91.70%
GAN	91.55%	92.06%	91.55%	91.51%
Our	**98.27%**	**98.34%**	**98.27%**	**98.27%**

The best classification results might result from the reasons below. In our 1D-CNN model, four feature extraction basic blocks with different sizes of convolutional kernels from large to small are included, thus semantic information of the high-level features and spatial information of the low-level features in spectral can be extracted, which greatly enriches the information contained in features [36]. In addition, the weighted cross-entropy loss function is used to assign different weights to different categories of losses, which can decrease the bias in model learning due to dataset imbalance between different groups and improve classification accuracy in multi-classification task [43]. Besides, although the sample size is relatively small in our study, a data augmentation method of the random linear combination of three spectra in the same groups is employed to generate new SERS spectra, in which the diversity of spectra increased, while the original distribution of each spectrum can be maintained, which can effectively avoid the overfitting problem.

### Spectral interpretable analysis

**Fig. 6:**
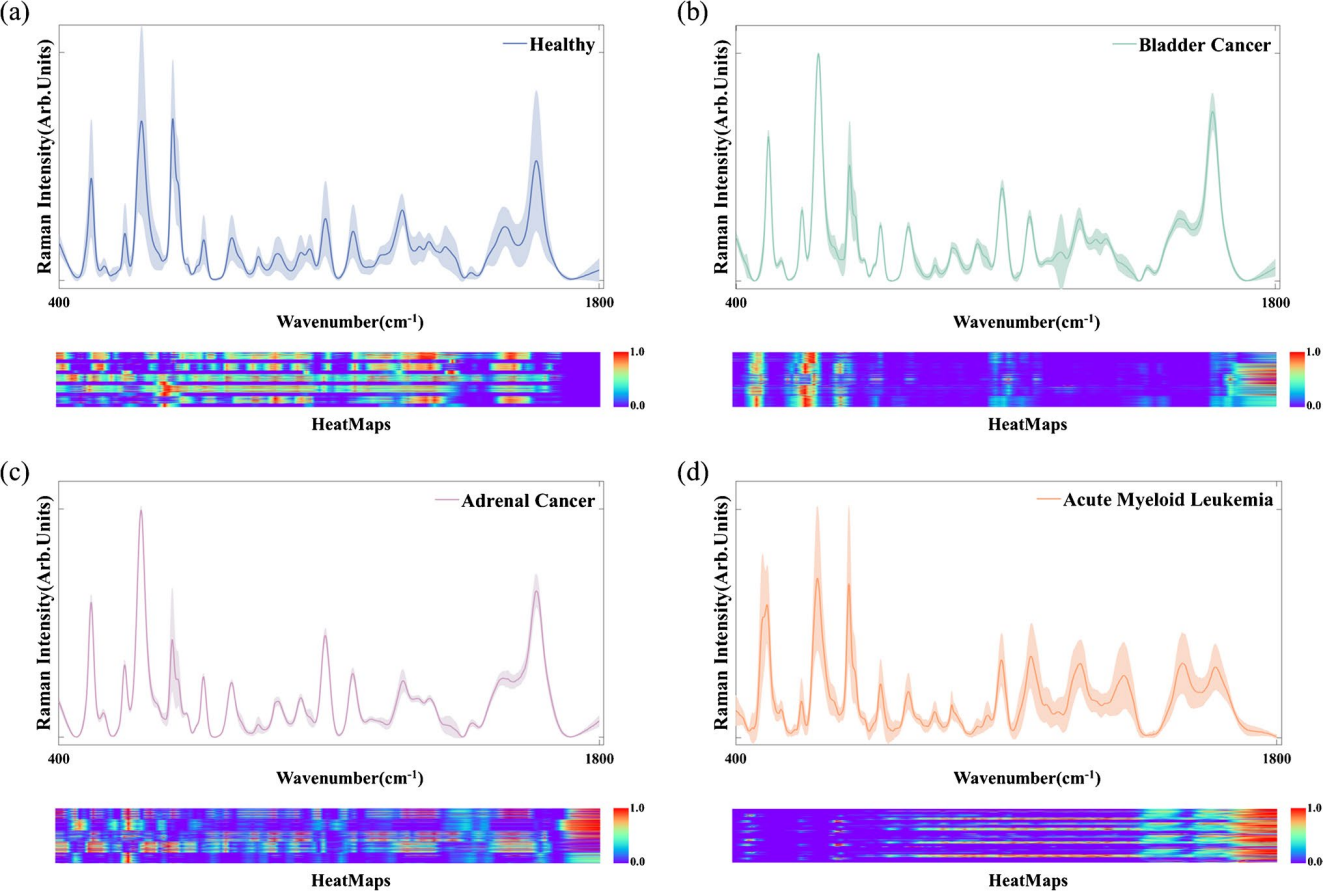
Serum SERS spectra and their heatmaps (i.e., the contribution degree of different wavenumbers for the final classification making) in **a** healthy controls; **b** bladder cancer; **c** adrenal cancer; **d** acute myeloid leukemia groups

Based on the Grad-CAM method, the contribution degree of different wavenumbers for the final classification making is shown as heatmaps in Fig. 6a–d, respectively. The horizontal dimension represents different wavenumbers (i.e., different biochemical substances corresponding to different Raman peaks at certain wavenumbers). The color represents the degree of contribution. From Fig. 6, it can be seen that the distribution of contribution degree varies greatly among four different groups. Although there are some variations in the intra-group, the heatmaps of each group basically follow strap distribution, indicating that the major contributions originated from consistent metabolic changes within each group.

**Fig. 7:**
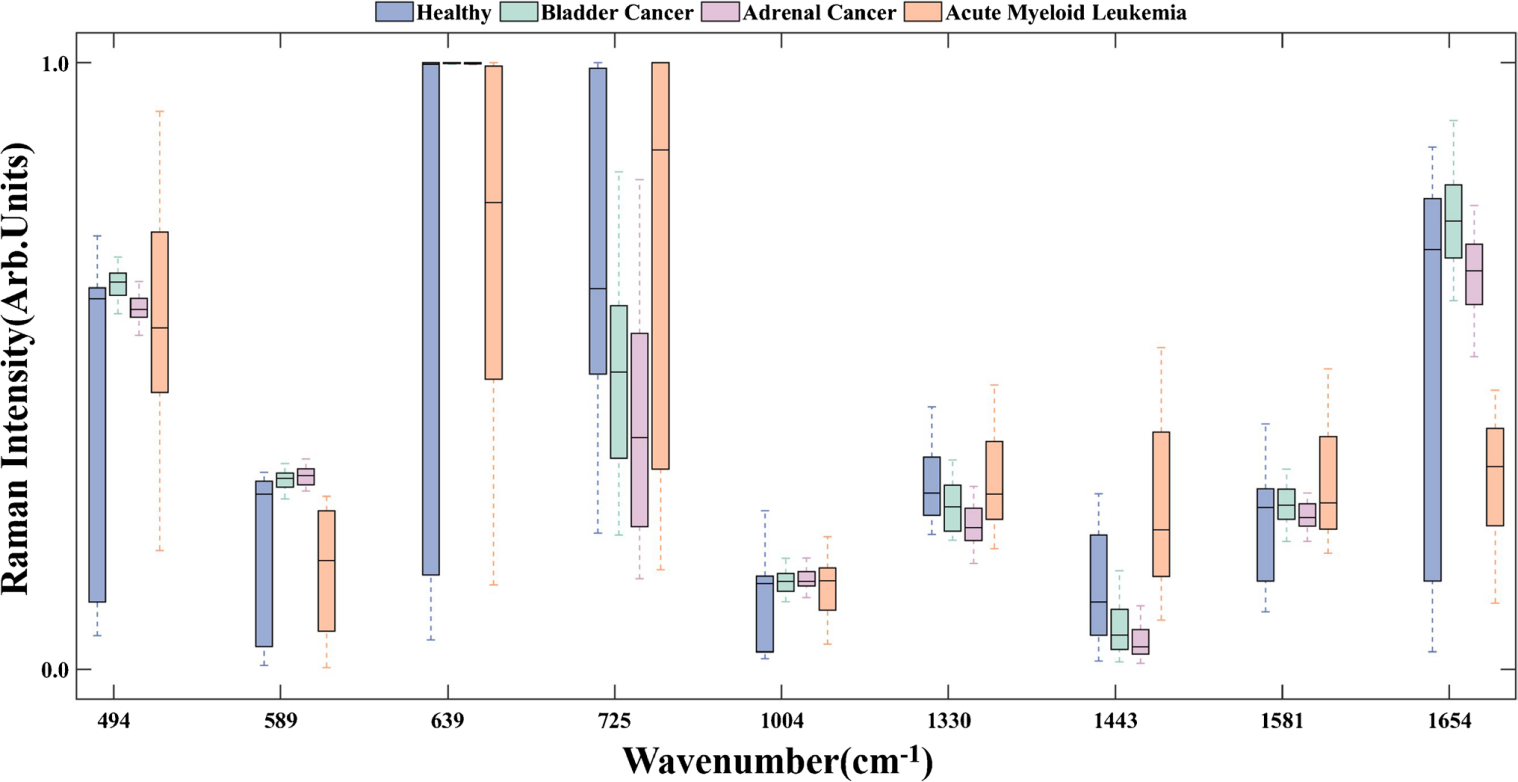
Boxplot of Raman intensities at peaks with the most significant contributions (i.e., contribution ≥ 10%) in healthy controls, bladder cancer, adrenal cancer, and acute myeloid leukemia groups

Table 3 shows the contribution of the prominent SERS peaks corresponding to biochemical substances for the final classification making in different groups. Furtherly, the boxplot of Raman intensities at peaks with outstanding contributions (i.e., contribution  ≥ 10%) in Fig. 7 shows the statistical differences among different groups. Thus, those SERS peaks with large contributions can indicate the most potential biomarkers in different cancers diagnosis.

**Table 3:** The contributions of biochemical substances on final classification making in healthy controls, bladder cancer, adrenal cancer and acute myeloid leukemia groups, respectively, in which the contributions larger than 10% are shown in bold

Peak position (cm^−1^)	Assignments	Healthy	Bladder cancer	Adrenal cancer	Acute myeloid Leukemia
494	Ring vibration, cellulose, guanine, L-arginine	2.51%	**10.40%**	4.02%	0.47%
531	Cholesterol ester	4.82%	0.59%	4.47%	0.02%
589	Ascorbic acid, amide-VI	2.06%	**15.25%**	4.79%	0.44%
639	C-S stretching vibration, L-tyrosine, lactose	1.85%	**30.80%**	6.67%	0.14%
725	C-H bending vibration, adenine, coenzyme A	5.08%	**10.84%**	5.65%	0.80%
812	C-C-O stretching vibration, L-serine, glutathione	2.79%	4.27%	1.12%	0.65%
887	C-O-H bending vibration, glutathione, D-( +)-galactosamine	6.19%	2.07%	4.02%	2.20%
959	α-Helix, L-proline, L-valine	6.15%	0.25%	4.60%	4.65%
1004	C-C symmetric stretch, phenylalanine	**10.13%**	0.46%	7.07%	4.39%
1073	C-N stretching vibration, collagen	7.94%	0.35%	6.39%	3.85%
1093	C-N stretching vibration, D-mannos	4.06%	1.26%	2.47%	4.47%
1135	C-N stretching vibration, D-mannos	3.46%	8.66%	3.65%	4.35%
1206	Ring vibration, L-tryptophan, phenylalanine	4.66%	2.83%	3.81%	7.11%
1330	C-H stretching vibration, nucleic acid bases	**12.29%**	0.89%	9.59%	**11.69%**
1443	CH2 bending vibration, glycine, L-proline, stearic acid	6.83%	0.86%	6.13%	**14.22%**
1581	C = C bending vibration, phenylalanine, acetoacetate, riboflavin	**15.90%**	1.08%	**16.53%**	**13.49%**
1654	C = O stretching vibration, phospholipids, amide-I, α-Helix	3.29%	9.15%	9.01%	**27.05%**

For bladder cancer, Raman peaks of 494, 589, 639, and 725 cm^−1^ hold the most significant contributions (i.e., contribution  ≥ 10%) on the final classification making. Among them, 30.8% of contributions at the Raman peak of 693 cm^−1^ indicate that L-tyrosine is the most important biomarker to distinguish bladder cancer from healthy controls and the other two kinds of cancer patients. Moreover, levels of L-tyrosine increase in bladder cancer patients compared to that in healthy controls, which is the same finding as previous studies [50–52]. L-tyrosine is an aromatic amino acid that can be converted into a variety of metabolites through glucose metabolism or ketone metabolism pathways, such as L-dopamine [53] and dopaquinone [52]. When bladder cancer occurs, tumor cells require abundant nutrients and energy during abnormally vigorous growth and proliferation, resulting in disorders of glucose and amino acid metabolism, which might lead to the increase of L-tyrosine in blood serum.

For adrenal cancer, Raman peaks of 1581 cm^−1^ hold the most significant contributions to the final classification making, which indicates that acetoacetate and riboflavin are the most important biomarkers to distinguish bladder cancer from the other groups. In addition, compared to healthy controls, acetoacetate and riboflavin decrease in the blood serum of adrenal cancer patients. As an essential endocrine organ, the adrenal glands produce various hormones, such as epinephrine, norepinephrine, glucocorticoids, and salt corticoids, which can regulate the processes of glucose, protein, and lipid metabolism. Acetoacetate can be synthesized from acetyl coenzyme A produced by lipolysis [54]. Abnormal hormone secretion in adrenal cancer patients may disrupt their lipid metabolism, which may cause decreases of acetoacetate in blood serum. Riboflavin is usually present in biological fluids and tissues as coenzymes [55]. Riboflavin in the body is first catalyzed by riboflavin kinase to produce flavin mononucleotide (FMN), which in turn is catalyzed by FMN adenylyltransferase to produce flavin adenine dinucleotide (FAD) [56]. FMN and FAD are essential cofactors for numerous enzymes and can bind to related enzyme proteins to produce various flavoproteins, thus participating in the body’s biological oxidation and energy metabolism processes [57]. Therefore, an abnormal metabolic environment in adrenal cancer patients may disturb the riboflavin transport process, resulting in a decreasing riboflavin level in the blood.

For acute myeloid leukemia, Raman peaks of 1330, 1443, 1581, and 1654 cm^−1^ hold the most significant contributions (i.e., contribution  ≥ 10%) on final classification making. Among them, 27.05% of contributions at 1654 cm^−1^ indicate that phospholipids, amide-I, and α-Helix are the most important biomarkers to distinguish acute myeloid leukemia from the other groups. Furthermore, the levels of phospholipid, amide-I, and α-Helix in blood were lower in the acute myeloid leukemia groups than that in the healthy controls. These findings are identical to other studies of González-Solís et al. [49], Bai et al. [58], and Kuliszkiewicz-Janus et al. [59] Phospholipids are an essential part of biological membranes [60]; the increasing proliferation of malignant cells in acute myeloid leukemia patients requires synthesizing large amounts of cell membranes [61], which may be responsible for the decrease of phospholipids in blood. To our knowledge, abnormal mitochondrial metabolism is an important mechanism for the occurrence of acute myeloid leukemia. Sirtuins 3 (SIRT3) is a type of mitochondrial deacetylase in vivo that plays an important role in the regulation of the mitochondrial metabolic function of cells [62], and some studies have shown that SIRT3 decreased in acute myeloid leukemia patients [63]. The abnormal expression of this enzyme may contribute to the disturbance of energy metabolism in acute myeloid leukemia patients, resulting disorder of protein synthesis and degradation.

In total, by the combination of label-free SERS and 1D-CNN method, high identification accuracy and quantitative contributions of biomolecules in different cancer groups can be achieved. Although there is a relatively small dataset in our study, we still believe that the proposed method can work well or even better for different cancer identification when the amounts of samples increase. In future studies, a large size of sample dataset and even including other cancers will be investigated to validate and confirm those conclusions.

## Conclusion

In this study, label-free serum SERS and 1D-CNN method were combined to identify the biochemical changes between healthy controls and different cancers (bladder cancer, adrenal cancer, and acute myeloid leukemia). By comparing the classification performance of our method with traditional machine learning methods as well as other classical deep learning models together, the best results were obtained by our method with an accuracy of 98.27%, precision of 98.34%, recall of 98.27%, and F1-score of 98.27%. Then, an intelligent and quantitative spectral interpretation analysis based on Grad-CAM was performed to objectively localize the specific Raman peaks corresponding to biomolecules responsible for the final identification making. The results indicated the most potential biomarkers were L-tyrosine to identify bladder cancer from others; acetoacetate and riboflavin to identify adrenal cancer from others; phospholipids, amide-I, and α-Helix to identify acute myeloid leukemia from others, which might provide an insight into the mechanism of intelligent diagnosis of different cancers based on serum SERS. Therefore, it demonstrated that label-free SERS combined with deep learning has great potential for the non-invasive, rapid, and reliable detection of different cancers, which may significantly improve the precise diagnosis in clinical.
